# Supplementary material for: BingleSeq: a user-friendly R package for bulk and single-cell RNA-Seq data analysis
Source: PeerJ. 2020 Dec 22;8:e10469. doi: 10.7717/peerj.10469 (PMC7761193; doi:10.7717/peerj.10469)
Supplement: Supplemental Information 1 [file peerj-08-10469-s001.docx]

**S1 Table**. Comparison between *BingleSeq*’s Bulk RNA-Seq pipeline and other similar applications.

| **Functionality** | ***BingleSeq*** | *DEapp* | *DEBrowser* | *Omics Playground* |
| --- | --- | --- | --- | --- |
| Filter Low Gene Counts | **✓** | **✓** | **✓** | **✓** |
| Batch-effect Correction | **✓** |  | **✓** | **✓** |
| Implements *DESeq2* | **✓** | **✓** | **✓** | **✓** |
| Implements *EdgeR* | **✓** | **✓** | **✓** | **✓** |
| Implements *limma* | **✓** | **✓** | **✓** | **✓** |
| Additional DE methods |  |  |  | **✓** |
| PCA plot | **✓** | **✓** | **✓** | **✓** |
| Summary Barchart | **✓** |  |  | **✓** |
| MA plot | **✓** |  | **✓** | **✓** |
| Volcano plot | **✓** | **✓** | **✓** | **✓** |
| All to all scatter plot |  |  | **✓** | **✓** |
| Heatmap | **✓** |  | **✓** | **✓** |
| Interquartile Range plot |  | **✓** | **✓** | **✓** |
