# Supplementary material for: BingleSeq: a user-friendly R package for bulk and single-cell RNA-Seq data analysis
Source: PeerJ. 2020 Dec 22;8:e10469. doi: 10.7717/peerj.10469 (PMC7761193; doi:10.7717/peerj.10469)
Supplement: Supplemental Information 2 [file peerj-08-10469-s002.docx]

| **Functionality** | ***BingleSeq*** | *singleCellTK* | *SeuratWizard* | *ASAP* | *Omics Playground* |
| --- | --- | --- | --- | --- | --- |
| Filtering/Normalization/Scaling | **✓** | **✓** | **✓** | **✓** | **✓** |
| Batch effect correction |  | **✓** |  |  | **✓** |
| Pre-cluster Noise Filtering | **✓** |  | **✓** | **✓** |  |
| PCA Dimension Reduction | **✓** | **✓** | **✓** | **✓** | **✓** |
| tSNE Dimension Reduction | **✓** | **✓** | **✓** | **✓** | **✓** |
| UMAP Dimension Reduction |  | **✓** |  |  |  |
| Multiple Clustering Algorithms | **✓** | **✓** | **✓** | **✓** |  |
| Estimate cluster number | **✓** | **✓** |  | **✓** | **✓** |
| Define Cluster Number | **✓** | **✓** |  | **✓** |  |
| Trajectory (Pseudotime) |  | **✓** |  | **✓** |  |
| DGE/Biomarker Visualization | **✓** | **✓** | **✓** |  | **✓** |
| Differential Expression | **✓** | **✓** | **✓** | **✓** | **✓** |
