# Supplementary material for: BingleSeq: a user-friendly R package for bulk and single-cell RNA-Seq data analysis
Source: PeerJ. 2020 Dec 22;8:e10469. doi: 10.7717/peerj.10469 (PMC7761193; doi:10.7717/peerj.10469)
Supplement: Supplemental Information 3 [file peerj-08-10469-s003.docx]

| **Functionality** | ***BingleSeq*** | *singleCellTK* | *DEapp* | *DEBrowser* | *SeuratWizard* | *ASAP* | *Omics Playground* |
| --- | --- | --- | --- | --- | --- | --- | --- |
| Customizable plots | **✓** |  |  | **✓** | **✓** | **✓** | **✓** |
| Interactive plots |  |  |  | **✓** | **✓** | **✓** | **✓** |
| GO Term Analysis | **✓** |  |  | **✓** | **✓** | **✓** | **✓** |
| GO Term Search | **✓** |  |  |  |  |  |  |
| KEGG Pathway Analysis | **✓** |  |  | **✓** | **✓** | **✓** | **✓** |
| Transcription Factor footprint-based analysis | **✓** |  |  |  |  |  |  |
| Pathway footprint-based analysis | **✓** |  |  |  |  |  |  |
| Disease-related Analysis |  |  |  | **✓** | **✓** |  |  |
| Oncogenic Signatures |  |  |  |  |  | **✓** |  |
| Copy number variation analysis |  |  |  |  |  |  | **✓** |
| Interactive Network Visualizations |  |  |  |  |  |  | **✓** |
| DE Method Comparison | **✓** | **✓** | **✓** |  |  |  | **✓** |
| DE Rank-based Consensus | **✓** |  |  |  |  |  |  |
| Intersection Analysis |  |  |  |  |  |  | **✓** |
| Complex Experimental Design | **✓** | **✓** | **✓** | **✓** | **✓** |  | **✓** |
| Download data and plots | **✓** |  |  | **✓** | **✓** | **✓** | **✓** |
| Inbuilt scRNA-Seq Pipeline | **✓** |  |  |  |  | **✓** | **✓** |
| Proteomics Analysis |  |  |  |  |  |  | **✓** |
| Manual cluster annotation |  |  |  |  |  | **✓** |  |
| Development | R/shiny | R/shiny | R/shiny | R/shiny | R/shiny | Multi-language | R/shiny |
