# Supplementary figures and images for: BingleSeq: a user-friendly R package for bulk and single-cell RNA-Seq data analysis
Source: PeerJ. 2020 Dec 22;8:e10469. doi: 10.7717/peerj.10469 (PMC7761193; doi:10.7717/peerj.10469)

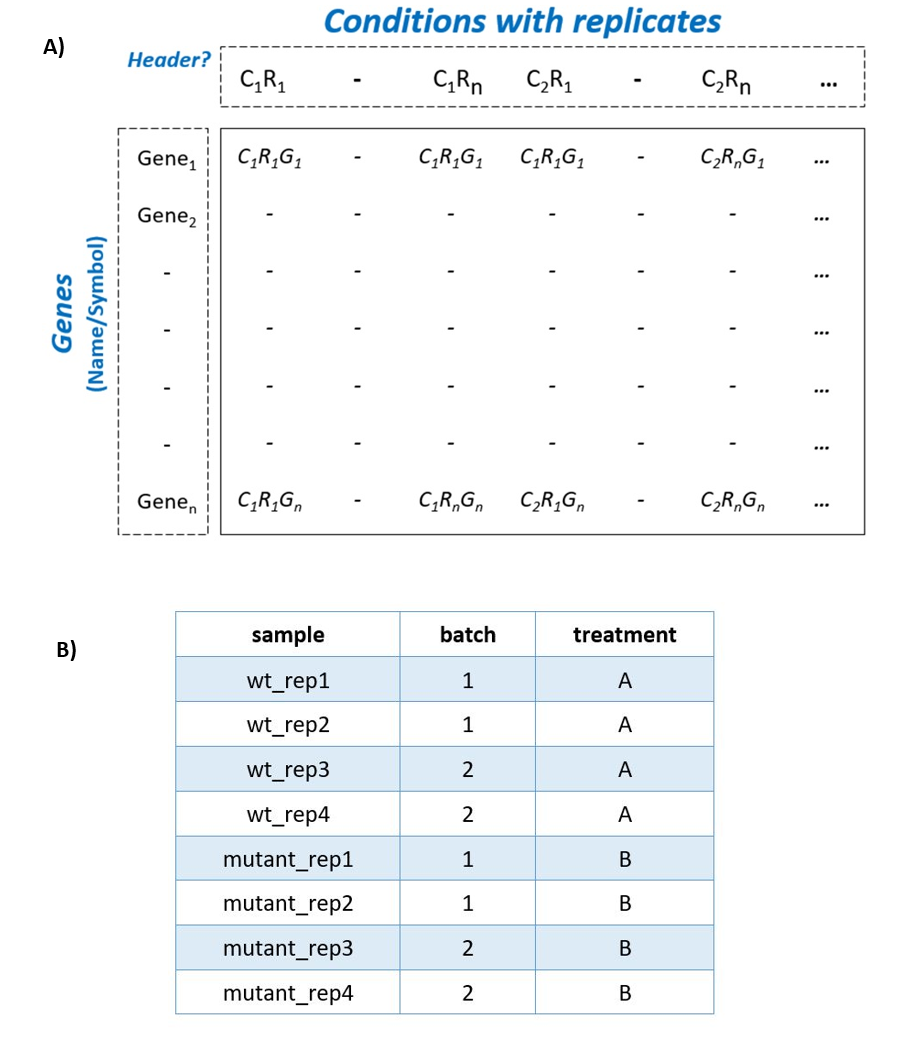

Supplement: Supplemental Information 4 [file peerj-08-10469-s004.png]

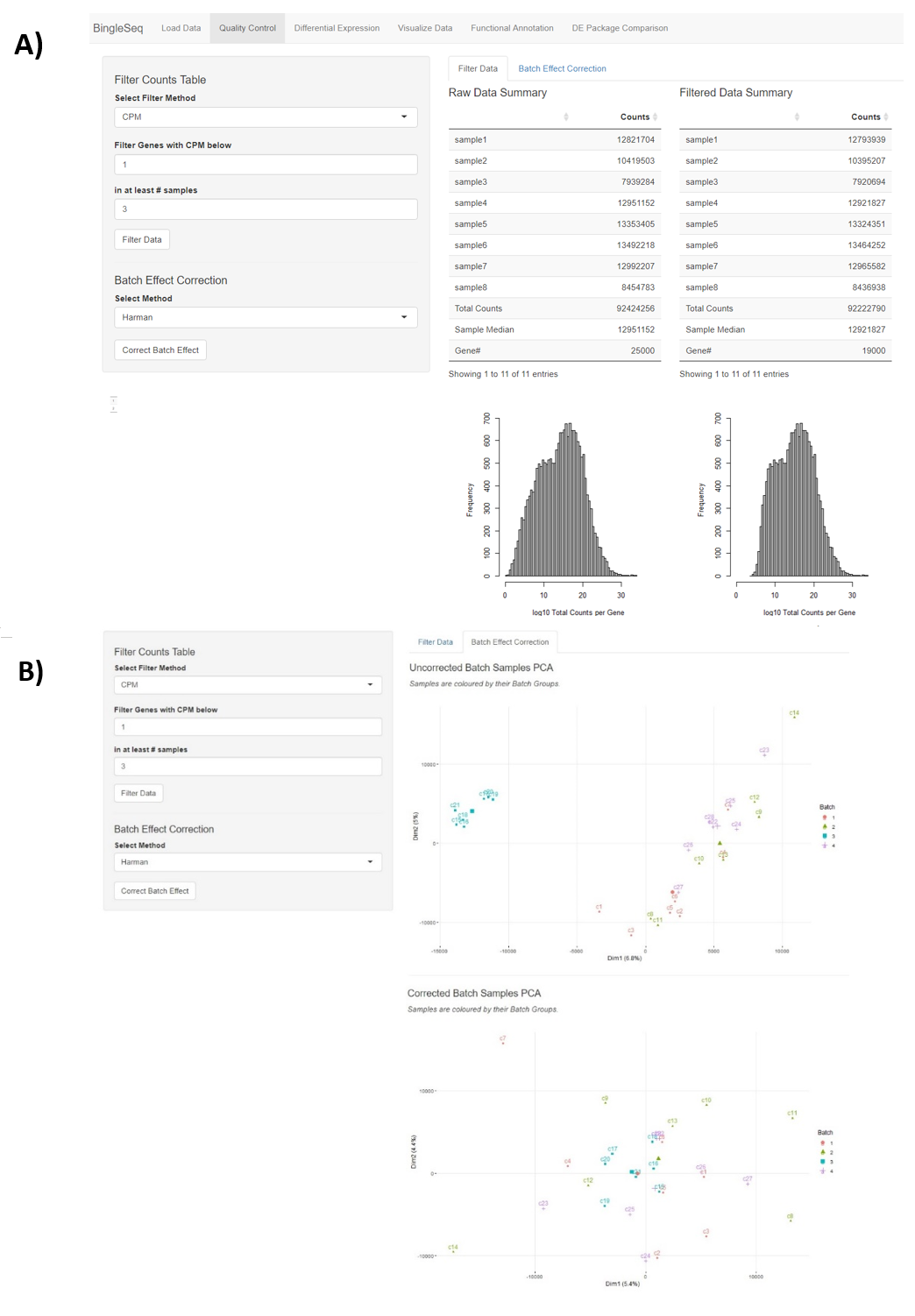

Supplement: Supplemental Information 5 [file peerj-08-10469-s005.png]

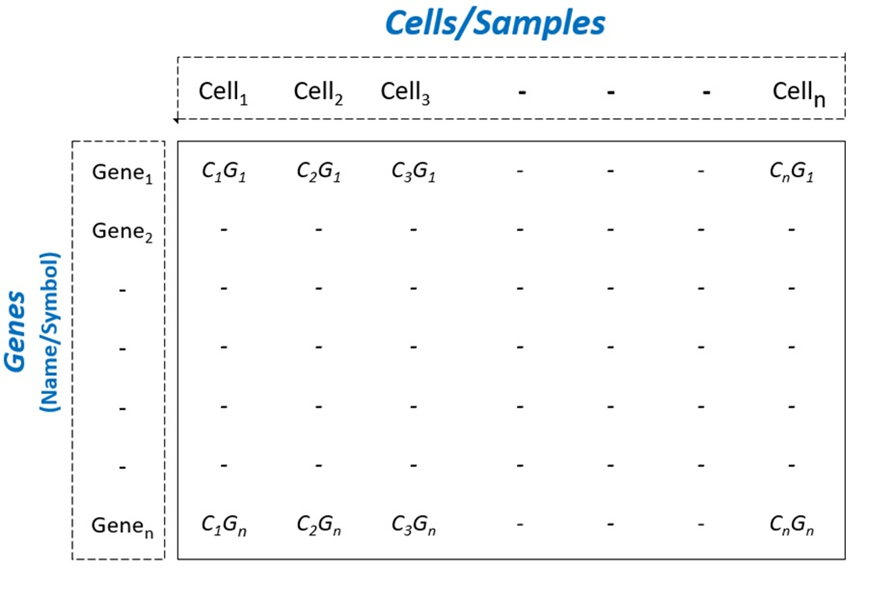

Supplement: Supplemental Information 6 [file peerj-08-10469-s006.png]

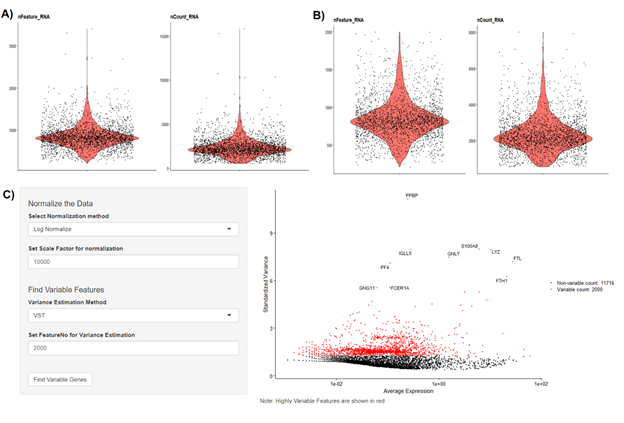

Supplement: Supplemental Information 7 [file peerj-08-10469-s007.png]

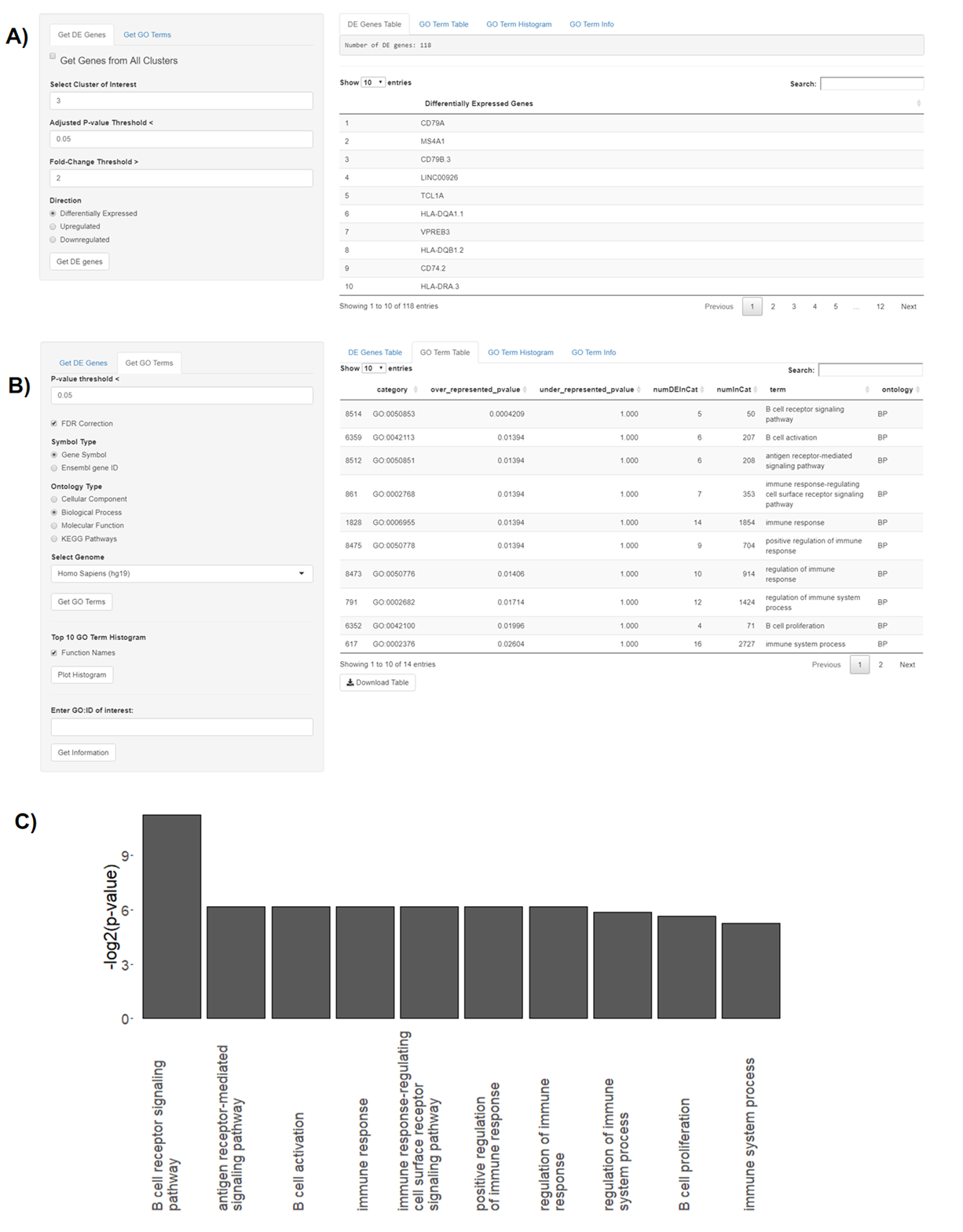

Supplement: Supplemental Information 8 [file peerj-08-10469-s008.png]

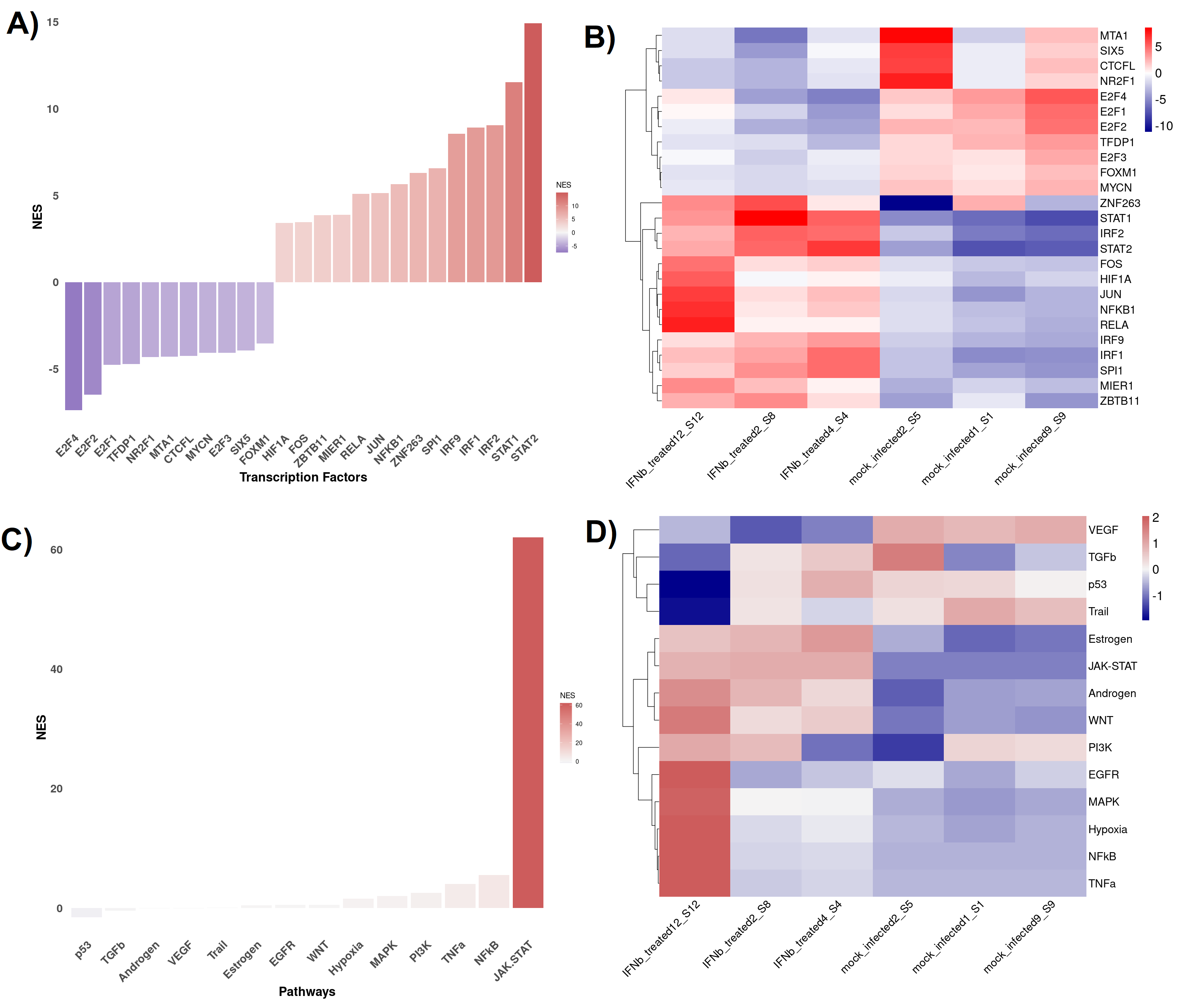

Supplement: Supplemental Information 9 [file peerj-08-10469-s009.png]

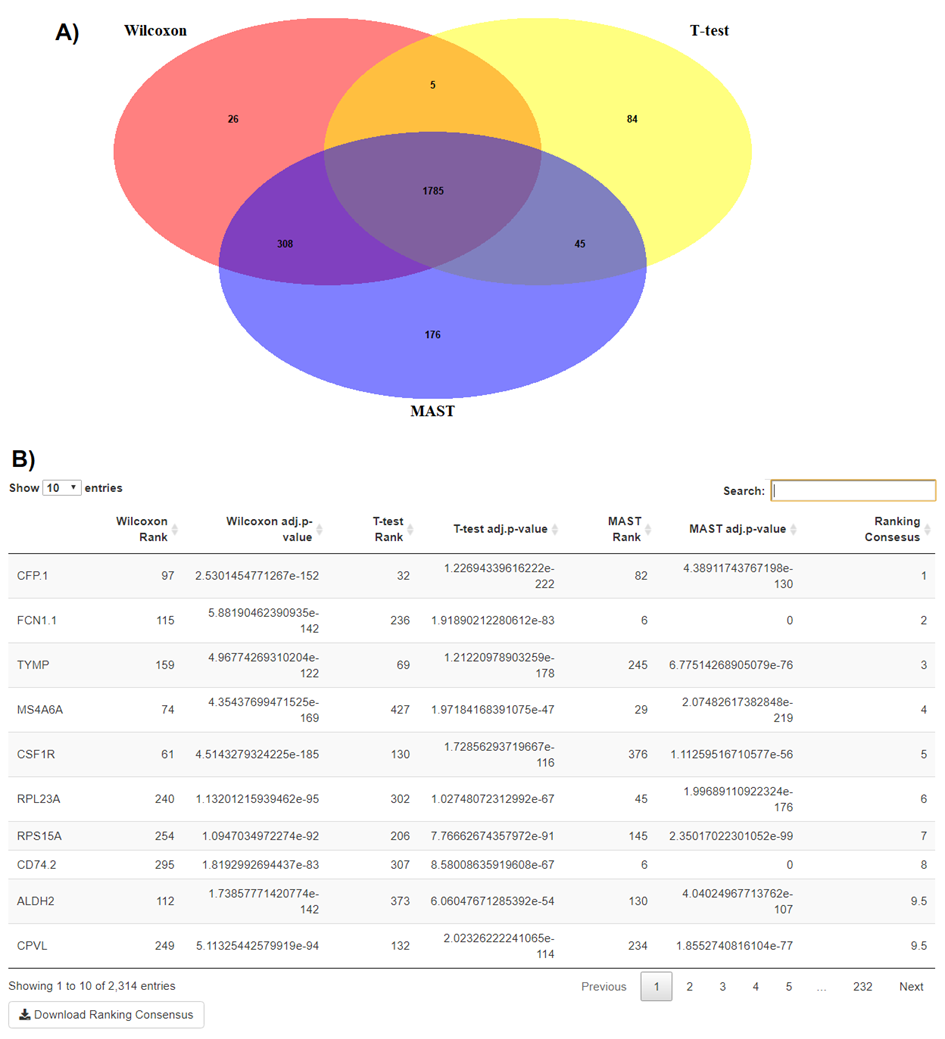

Supplement: Supplemental Information 10 [file peerj-08-10469-s010.png]
