# Supplementary material for: BingleSeq: a user-friendly R package for bulk and single-cell RNA-Seq data analysis
Source: PeerJ. 2020 Dec 22;8:e10469. doi: 10.7717/peerj.10469 (PMC7761193; doi:10.7717/peerj.10469)
Supplement: Supplemental Information 11 [file peerj-08-10469-s011.pdf]

Bulk RNA-Seq Pipeline Evaluation

Simulated data set evaluation

Evaluation of BingleSeq's Bulk RNA-Seq pipeline was first performed using synthetic data generated with compcodeR package. compcodeR was used to simulate 6 two-condition datasets each with 20,000 features and a sequencing depth of 1e+07, varying between 0.7x and 1.4x this depth. There were no simulated outliers and of these 20,000 genes, 1000 were upregulated and 1000 downregulated. The datasets were divided in two groups that differed in the minimal differential expression strength of true positive DEGs. DEGs from the first and second groups had a minimal differential expression strength of 2 and 4, respectively. Each of these groups was composed of 3 datasets in total with 3, 6, and 12 replicates per condition.

BingleSeq was then used to generate DE results for each of the simulated datasets using the DE packages incorporated within its Bulk RNA-Seq pipeline. The obtained results were then assessed, and the sensitivity and specificity of the packages are shown in **fig. 1**. The lowest sensitivity was observed in the dataset with 3 replicates and effect size of 2, while the highest sensitivity was seen in the dataset with 12 replicates and DEGs with an effect size of 4 (**fig. 1A**). Therefore, as anticipated, sensitivity increased with both the number of replicates and effect size. Furthermore, the packages were rather conservative and relatively few DEGs were false positives, regardless of the dataset; hence, each package had high specificity (**fig. 1B**). These results confirm that the DE pipelines implemented in BingleSeq are capable of producing adequate DE analysis results.

Note that different sequencing depths were accounted for using the package-specific default methods for DESeq2 and edgeR, and voom and TMM normalization for limma. Similarly, the default test methods for each package were used for the analysis of the synthetic data set.

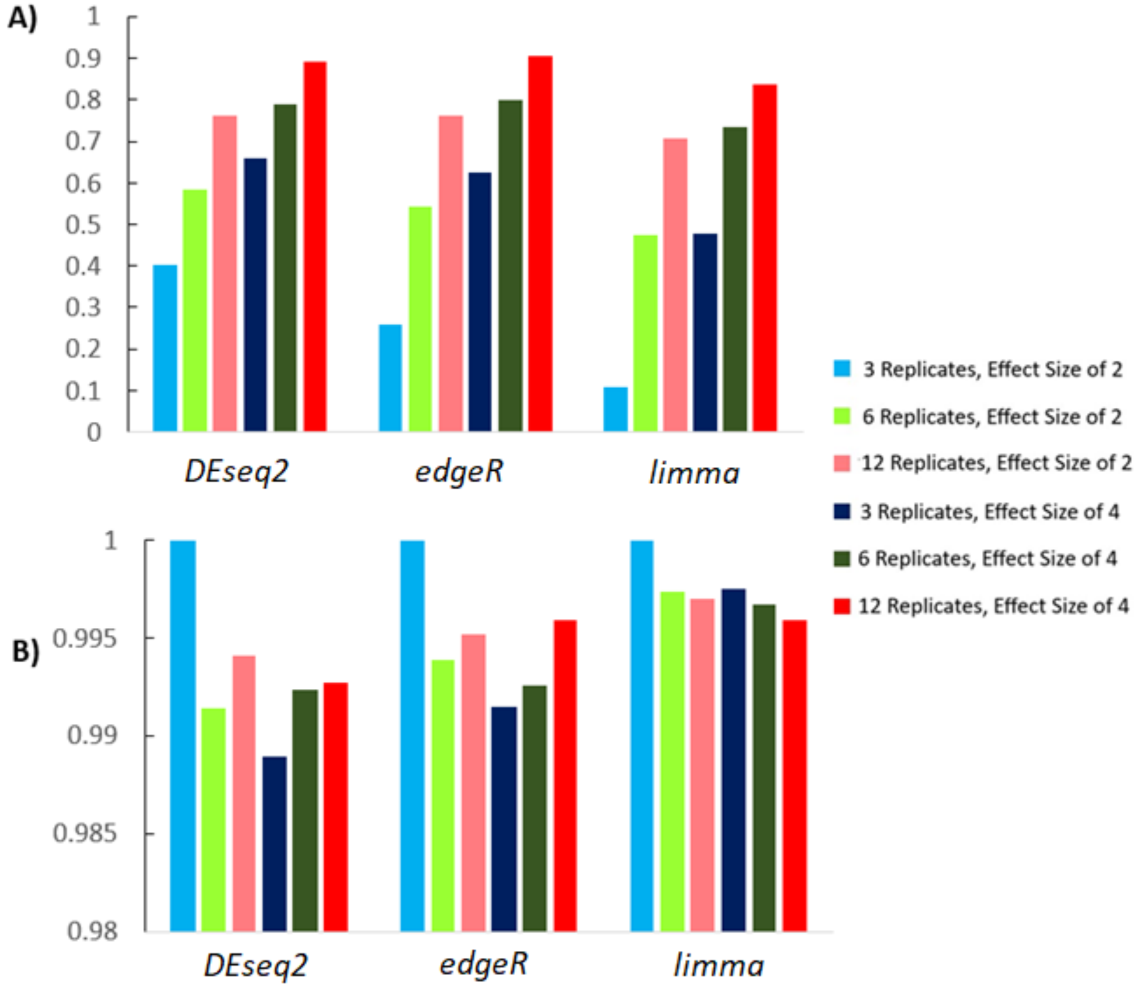

Figure 1. *Statistical measures of performance (Sensitivity* ***A)*** *and Specificity* ***B)****) of DESeq2, edgeR, and limma as observed for each of the simulated datasets. See* ***Table 1*** *for tables with the obtained results.*

***Table 1**. Differential Analysis results for the simulated datasets obtained by **A)** DESeq2, **B)** edgeR, and **C)** limma pipelines as implemented in BingleSeq. Note that R stands for replicates and ES for effect size.*

A) *DE analysis results obtained using DESeq2.*

	3R; ES2	6R; ES2	12R; ES2	3R; ES4	6R; ES4	12R; ES6
DEGs	894	1319	1629	1517	1718	1911
True Positives	744	1164	1522	1318	1580	1780
False Positives	0	155	107	199	138	131
False Negatives	1106	836	478	682	420	220
True Negatives	18000	17845	17893	17801	17862	17869
Sensitivity	0.402	0.582	0.761	0.659	0.790	0.890
Specificity	1.000	0.991	0.994	0.989	0.992	0.993

B) *DE analysis results obtained using edgeR.*

	3R; ES2	6R; ES2	12R; ES2	3R; ES4	6R; ES4	12R; ES6
DEGs	575	1192	1606	1403	1730	1885
True Positives	494	1082	1519	1249	1596	1811
False Positives	0	110	87	154	134	74
False Negatives	1425	918	481	751	404	189
True Negatives	18000	17890	17913	17846	17866	17926
Sensitivity	0.257	0.541	0.760	0.625	0.798	0.906
Specificity	1.000	0.994	0.995	0.991	0.993	0.996

C) *DE analysis results obtained using limma*.

	3R; ES2	6R; ES2	12R; ES2	3R; ES4	6R; ES4	12R; ES6
DEGs	218	995	1467	1000	1525	1749
True Positives	213	947	1413	955	1465	1675
False Positives	0	48	54	45	60	74
False Negatives	1782	1053	587	1045	535	325
True Negatives	18000	17952	17946	17955	17940	17926
Sensitivity	0.107	0.474	0.707	0.478	0.733	0.838
Specificity	1.000	0.997	0.997	0.998	0.997	0.996

Real world data set evaluation

Following the evaluation using the synthetic data set, we used real world data (McFarlane et al., 2019) to showcase the applicability of BingleSeq's Bulk RNA-Seq pipeline. This dataset examined the differentially expressed genes (DEGs) between HSV-1 infected control and interferon B treatment.

First the data was filtered for genes with count per million (CPM) below 1 in at least 2 of the samples **(Fig. 2).**

A)

Raw Data Summary

	Counts
mock_infected1_S1	31982285
mock_infected2_S5	34742775
mock_infected9_S9	28853256
IFNb_treated12_S12	34376227
IFNb_treated2_S8	40171321
IFNb_treated4_S4	32451841
Total Counts	202577705
Sample Median	34376227
Gene#	58051

Showing 1 to 9 of 9 entries

Filtered Data Summary

	Counts
mock_infected1_S1	31923291
mock_infected2_S5	34682503
mock_infected9_S9	28804742
IFNb_treated12_S12	34321006
IFNb_treated2_S8	40107936
IFNb_treated4_S4	32402179
Total Counts	202241657
Sample Median	34321006
Gene#	13718

Showing 1 to 9 of 9 entries

B)

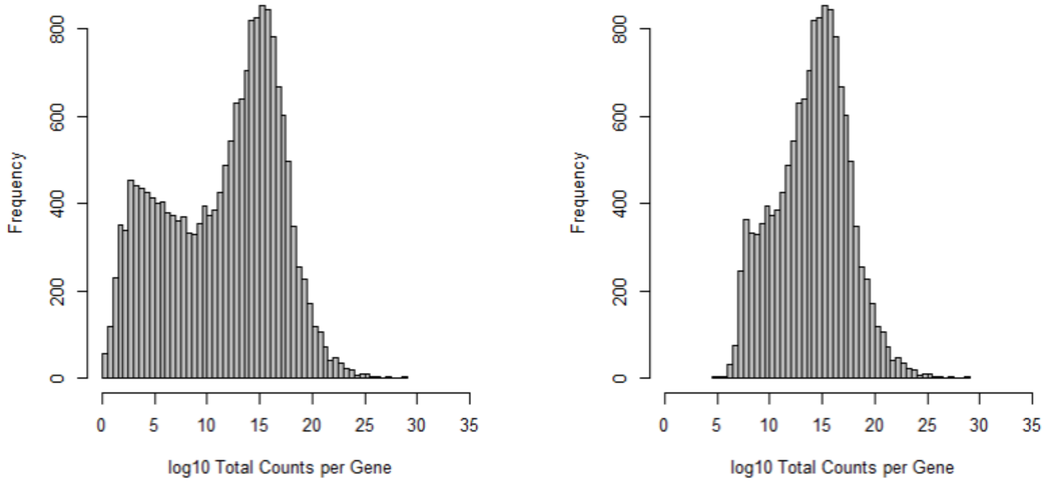

***Figure 2**. **A)** Count Data summaries before and after filtering with their corresponding **B)** gene frequency histograms.*

Following filtering differential expression (DE) testing was performed using limma with voom and TMM normalization. These results were then used for the subsequent visualization step of BingleSeq's Bulk RNA-Seq pipeline. In total, there were and 1053 up- and 1098 downregulated genes (**Fig. 3A**) with a visible distinction in the variation between the two conditions (**Fig. 3B**). Volcano (**Fig. 3C**) and MA (**Fig. 3D**) plots were then generated to represent the relationships between fold change (FC) versus significance and average expression, respectively.

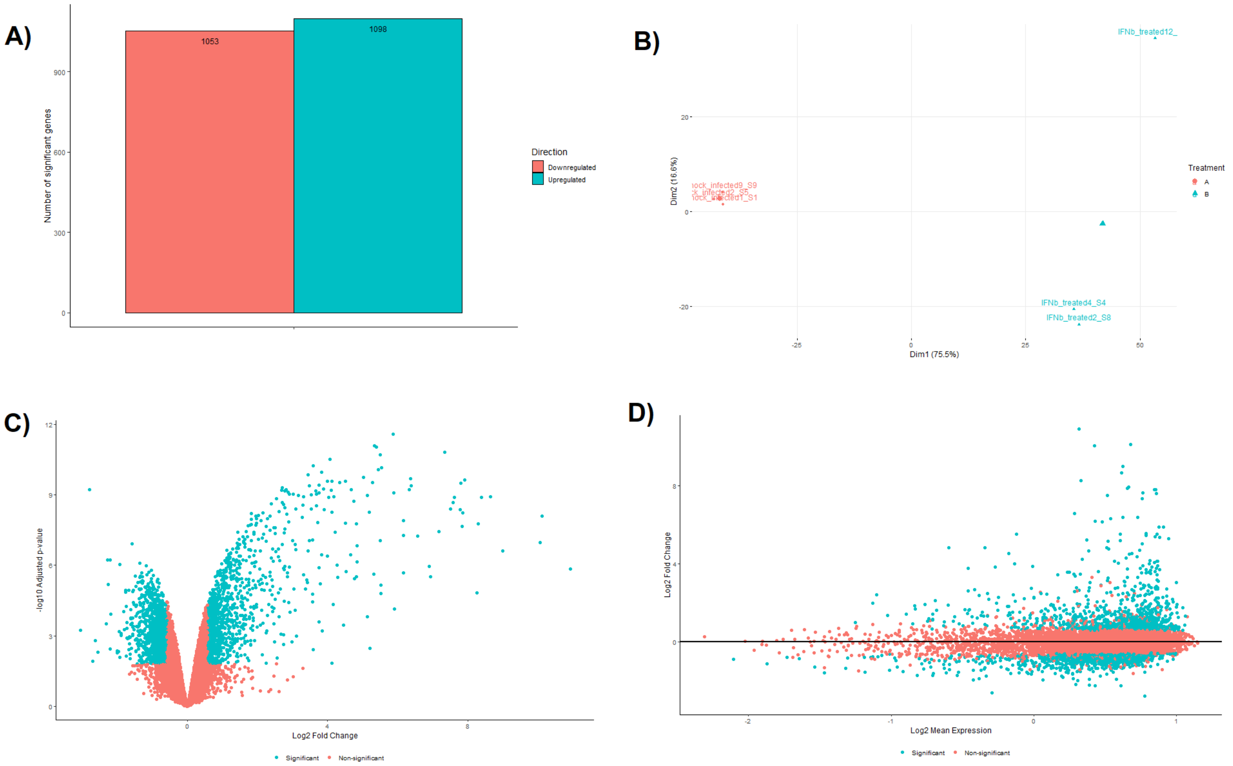

***Figure 3**. **A)** Barchart plot presenting the number of up- and downregulated genes **B)** PCA plot, **C)** Volcano plot, and **D)** MA plot.*

BingleSeq was also used to generate heatmaps for the top 20 up- and downregulated genes (**Fig. 4 A-B**) as well as for the functional gene annotation analysis of the DE results. The functional analysis revealed that 3 of the top 10 GO Biological Process terms were directly related to the cellular response to interferons. Thus, serving as further example of the applicability of the features implemented within our application. Note that each step was produced with DEGs filtered according to multiple-testing corrected p-value < 0.05 and log FC > 1.5.

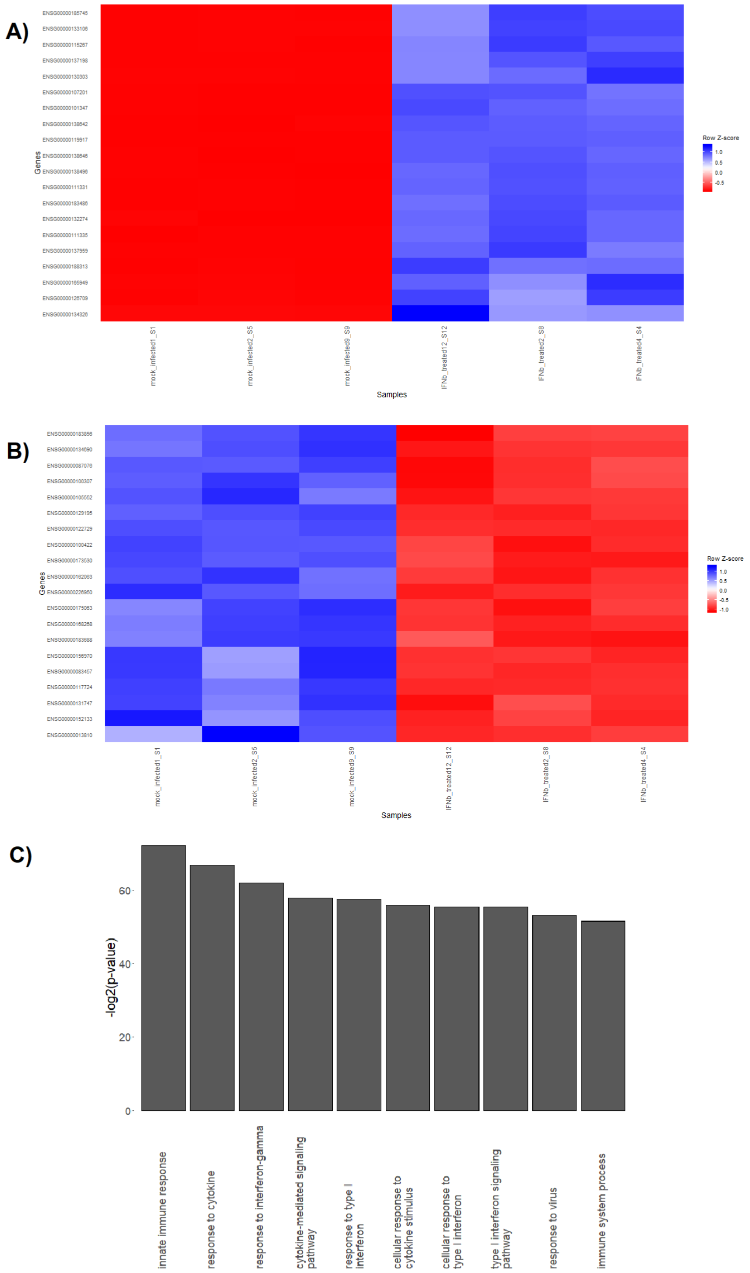

***Figure 4**. Heatmaps representing the top 20 **A)** up- and **B)** downregulated genes in the real- world data. **C)** Bar plot with showing the Top 10 most significant Biological Process Go terms.*

The agreement between DE packages was assessed by producing a Venn diagram with DEGs unfiltered by FC. The Venn diagram showed a considerable agreement between all 3 packages with DESeq2 and limma showing a slightly higher overlap in their results (**Fig. 5A**). Finally, the rank-based consensus table (**Fig. 5B**) showed considerable agreement in the ranking of the top 10 most significant genes, and it could also be used as an interactive tool that could be used to provide further confidence in specific genes of interest.

A)

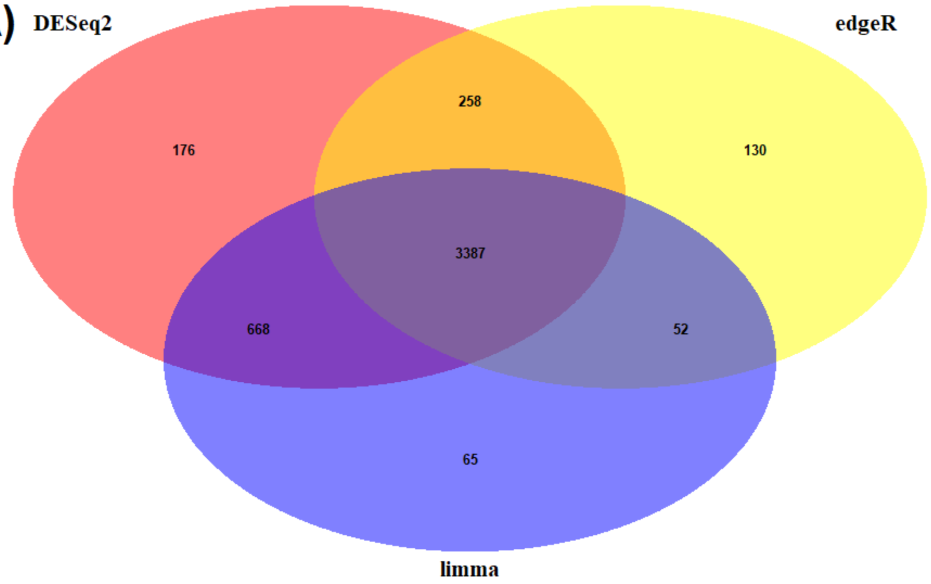

B)

Show 10 entries

Search:

	edgeR Rank	edgeR adj.p-value	limma Rank	limma adj.p-value	DESeq2 Rank	DESeq2 adj.p-value	Ranking Consesus
ENSG00000183486	1	9.16556584892308e-301	4.5	5.03881799032289e-8	9	0	1
ENSG00000119917	5	5.3094789581713e-250	1	3.32498473069603e-8	9	0	2
ENSG00000107201	9	2.54644982000429e-219	2.5	4.05945330173205e-8	9	0	3
ENSG00000111331	12	2.55002661384474e-214	2.5	4.05945330173205e-8	9	0	4
ENSG00000111335	13	5.09825449235143e-213	4.5	5.03881799032289e-8	9	0	5
ENSG00000137959	3	2.57292177332505e-288	16.5	2.06043462632089e-7	9	0	6
ENSG00000138646	8	1.27523170607536e-228	16.5	2.06043462632089e-7	9	0	7
ENSG00000115267	16	1.87859083393152e-209	9	1.1905802584263e-7	9	0	8
ENSG00000138642	18	1.49283520021086e-205	8	1.14784524067696e-7	9	0	9
ENSG00000133106	19	3.60431133203377e-189	12	1.9354154233462e-7	9	0	10

***Figure 5**. **A)** Venn diagram showing the overlap of DEGs obtained by the scRNA-Seq pipeline for the real-world data set. **B)** An interactive rank-based consensus table showing the top 10 highest ranked genes generated with the same data.*
